# Methods for exploring the faecal microbiome of premature infants: a review

**DOI:** 10.1186/s40748-021-00131-9

**Published:** 2021-03-08

**Authors:** Jacob A. F. Westaway, Roger Huerlimann, Catherine M. Miller, Yoga Kandasamy, Robert Norton, Donna Rudd

**Affiliations:** 1 James Cook University, 1 McGregor Road, Smithfield, QLD 4878 Australia; 2 James Cook University, 1 James Cook Dr, Douglas, QLD 4811 Australia; 3 Townsville University Hospital, 100 Angus Smith Dr, Douglas, QLD 4814 Australia; 4 Pathology Queensland, 100 Angus Smith Dr, Douglas, QLD 4814 Australia

**Keywords:** Microbiome, Premature, Neonate, Dysbiosis

## Abstract

The premature infant gut microbiome plays an important part in infant health and development, and recognition of the implications of microbial dysbiosis in premature infants has prompted significant research into these issues. The approaches to designing investigations into microbial populations are many and varied, each with its own benefits and limitations. The technique used can influence results, contributing to heterogeneity across studies. This review aimed to describe the most common techniques used in researching the preterm infant microbiome, detailing their various limitations. The objective was to provide those entering the field with a broad understanding of available methodologies, so that the likely effects of their use can be factored into literature interpretation and future study design. We found that although many techniques are used for characterising the premature infant microbiome, 16S rRNA short amplicon sequencing is the most common. 16S rRNA short amplicon sequencing has several benefits, including high accuracy, discoverability and high throughput capacity. However, this technique has limitations. Each stage of the protocol offers opportunities for the injection of bias. Bias can contribute to variability between studies using 16S rRNA high throughout sequencing. Thus, we recommend that the interpretation of previous results and future study design be given careful consideration.

## Introduction

The premature infant gut microbiome has become an important, modifiable factor in the field of neonatal intensive care. Compared with infants born full-term, the characteristic microbiome of premature infants (born <37 weeks gestation) is dysbiotic: highly variable [1–3], low in diversity [4–6], low in common commensals [1, 6, 7], and harbouring more potential pathogens [8, 9]. This dysbiotic microbiome composition puts immune-compromised premature infants at an increased risk of acute and chronic disease, and developmental abnormalities [10–12]. Premature infants are also more likely to be born via caesarean section, be formula fed, receive antibiotics, and spend much of their early life in a clinical environment, all of which have the potential to exacerbate the microbial dysbiosis [1, 2, 13, 14].

Unfortunately, understanding this microbial composition is confounded by the diversity of investigative methods used. Methodologies for examining the microbiome are complex, technically challenging and vary between laboratories. Therefore, it is impossible to rule out protocol bias as a factor contributing to the variability seen between studies. In fact, a number of studies have demonstrated the role of methodological bias in influencing the outcomes of microbiome analysis [15, 16], thus contributing to significant heterogeneity in results between studies.

This review aimed to describe the most common techniques used in researching the preterm infant microbiome. We chose to focus on those studies investigating preterm infants, due to the explosion of interest into this area, although these techniques can also be applied to microbiome study of full-term infants. This magnified interest likely stems from the disproportionate health burden placed on preterm infants and its link to the microbiome. The objective of this review was to provide those entering the field, particularly those in neonatal clinical care, with a broad understanding of the different methods used, so that literature interpretation and future study design can be enhanced. This review identifies and describes the most commonly used methods for examining the premature infant’s microbiome, and maps this information against studies comparing efficacy of techniques. This process is designed to illuminate which techniques will be most appropriate for the examination of the microbiome of premature infants

## Methods

### Search and Eligibility Criteria

**Fig. 1:**
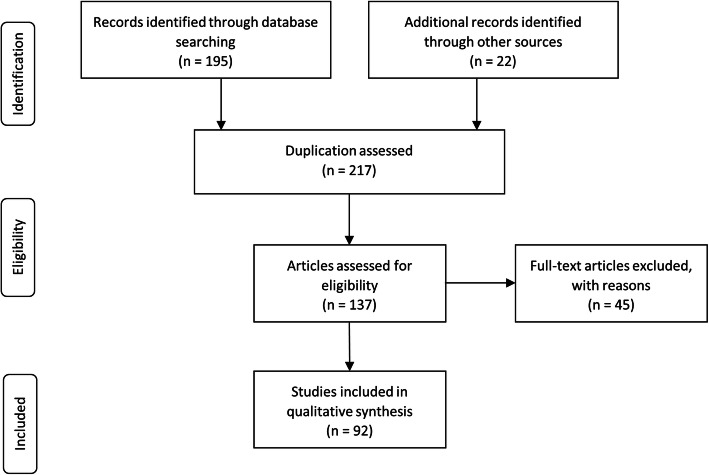
PRISMA flow diagram describing the process of study collection and inclusion

The PRISMA (Preferred Reporting Items for Systematic Reviews and Meta-Analyses) approach (Fig. 1), was taken to search for relevant literature up until August 2020. Studies investigating the premature microbiome were identified via searches in the SCOPUS and PubMed databases using the search terms; “Microbiota” AND “Infant” AND “Premature” AND “Faeces”. Journal articles describing a wide variety of study designs, sample sizes, interventions, comparators and outcomes were included in this review. Articles were excluded if they were not original studies, were case studies, were not in English, were unable to be accessed or did not specifically investigate the premature infant microbiome. Reviews found in the initial search were also used to locate other papers that addressed the review question.

### Data Collection Process

A standardised data collection protocol was established to extract all relevant information for qualitative analysis. Author, date of publication, aims/hypotheses, a summary of the methods, a summary of the findings and limitations were recorded. Methodology-specific information was also collected for primary techniques, secondary techniques, storage and DNA extraction. Emphasis was placed on 16S rRNA short amplicon sequencing, as this methodology was the most common primary technique, and further information was collected for target variable regions, platform, pipeline and reference databases.

## Results

**Fig. 2:**
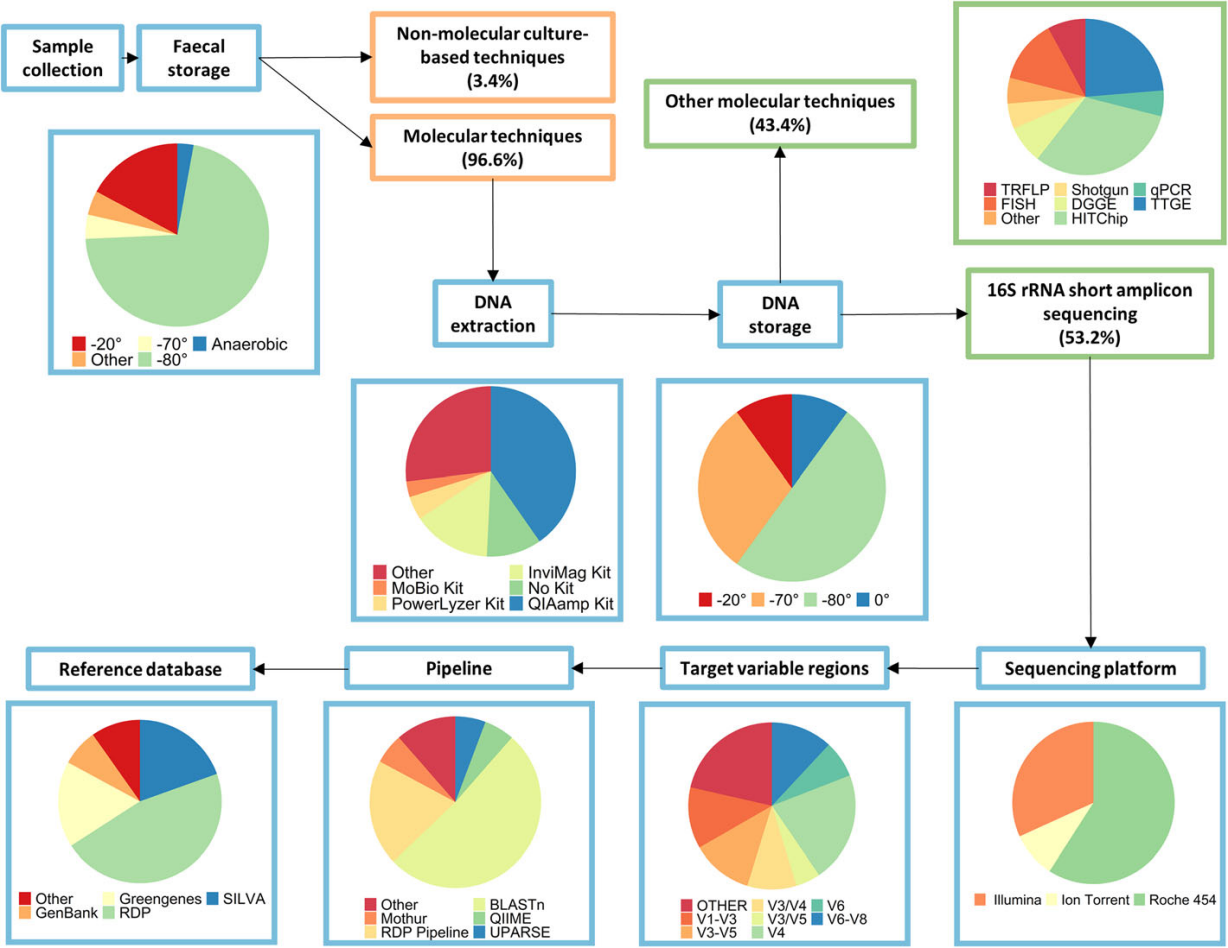
Flow diagram describing different workflows and proportions of techniques used for microbiome analysis in premature infants

The review of the literature explored the methodological diversity used in the study of the premature infant microbiome, and a summary of the major techniques used across the studies is presented in Fig. 2. The outcome of the systematic review is summarised in Fig. 1. Two hundred and seventeen articles were identified. Of these 137 articles remained after duplicates were removed, with a further 45 articles removed after assessing the full article for eligibility. A total of 92 articles were reviewed. There was a surprising lack of detail displayed in the methods section of many studies, despite there being several technical choices at each stage of the workflow with the potential to contribute to bias. The summary information is based only on the data that was made available.

### Primary Techniques

Thirteen techniques for characterising the preterm infant gut microbiome were identified (Fig. 2). A wide range of techniques have been used as the primary tool for microbial compositional analysis including:

- Traditional culture-based techniques [17–21],
- 16S rRNA short amplicon sequencing [3, 5–7, 13, 22–65],
- Shotgun metagenomics [2],
- Temperature gradient gel electrophoresis (TGGE) [9, 10, 66–72],
- Denaturing gradient gel electrophoresis (DGGE) [8, 73–76],
- HITChip [77, 78],
- Fluorescent In-Situ Hybridisation Analysis (FISH) [79, 80],
- Terminal restriction fragment length polymorphism (TRFLP) [81–83],
- Quantitative PCR (qPCR) [9, 44, 84–93],
- Long-read nanopore sequencing [94], and
- Random amplified polymorphic DNA/pulsed-field gel electrophoresis (RAPD/PFGE) [95].

Molecular techniques dominated, specifically 16S rRNA short amplicon sequencing, which made up 53.2% of primary techniques. qPCR was the second most commonly used technique, used in 13% of studies, with the remaining techniques being used by ≤10% of studies each. Traditional non-molecular techniques using selective and differential agar medium represented a very small fraction of the primary techniques used (5.4%), and a further four studies used culture techniques as a secondary method.

### Storage Conditions and Extraction Protocol on DNA for Molecular Techniques

Sample storage protocols were consistent across the molecular techniques. However, the DNA extraction techniques used were highly variable. Freezing at -80°C dominated storage methods for both faeces (71.4%) and DNA (50%), with non-freezing protocols only used in 2.9% of studies. DNA extraction was the area demonstrating the greatest variability, with 15 different methods utilised. The QIAamp DNA Stool Kit, a kit that combines heat, chemical and enzymatic lysis was the most commonly used (40.3%), with using no kit at all (14.9%) the second most common option. The PowerLyzer PowerSoil kit was third (10.4%), with the other twelve kits making up the remaining 34.4%.

### 16S rRNA Amplicon Sequencing – Specific Techniques

The most common molecular technique was 16S rRNA short amplicon sequencing. However, the methods used were highly variable in sequencing platforms, variable target regions, and pipelines. Roche 454 (57.8%) sequencing platform was the most commonly utilised of the four, with Illumina second (31.1%). The use of this platform has increased in recent years. V4 was the most common variable target region used (22.7%). However, there were sixteen unique combinations used across the 92 studies. Of the eight pipelines used, QIIME/QIIME2 (Quantitative Insights Into Microbial Ecology) [96] made up half of the pipelines used, with Mothur [97] a distant second (20%). The Ribosomal Database Project (46.3%) was the predominant reference database used, with SILVA (19.5%) and Greengenes (17.1%) being used in most of the remaining studies. Techniques specific to 16S rRNA short amplicon sequencing varied greatly, despite it being the most common method.

### Trends Over Time

Many outdated techniques and tools are being abandoned for newer, more robust methods. High throughput molecular techniques have become more commonly used over time, especially in 16S rRNA amplicon sequencing, which was first used in 2004. This upward trend in 16S rRNA amplicon sequencing is coupled with a decline in both fingerprinting- and culture-based techniques, with all culture-based studies occurring prior to 2015. There is also a trend towards the use of Illumina platforms and pipelines that use error modelling within 16S rRNA amplicon sequencing.

## Discussion

Techniques for examining the microbiome can be categorised into two main groups, molecular and non-molecular. Molecular techniques have become dominant due to their depth of analysis, speed and cost reduction. Nevertheless, there are several techniques to choose from even within molecular methodologies, and within a given molecular technique there is variation possible in protocols. This lack of consistency can contribute to the inconsistencies in results between studies into the premature microbiome.

### Culture Based Approaches

Few studies still rely solely on traditional non-molecular methods for microbiome characterisation, with the most recent study occurring in 2014. Non-molecular techniques are based on traditional microbiological methods that involve growing microbial communities on predetermined growth media under strict laboratory conditions designed to optimise growth. Methods vary depending on the type of micro-organisms present and downstream applications. Techniques include broth culture, enrichment and microbial identification. Examples of growth media include Luria Broth, also known as Lysogeny Broth [98], which is common for the cultivation of *Escherichia coli*, and selective agars such as blood, MacConkey [99] or Xylose Lysine Deoxycholate agar [100], which are specific for other taxa prevalent in the gastrointestinal tract. Microorganisms are placed in growth medium and left to grow under strict conditions, giving them time to grow into individual colonies. Colony morphology can then be used to determine specific taxa and colony counts, and are used for the calculation of concentrations and serial dilutions. These techniques are primarily used to identify specific microorganisms of interest due to their specificity, and employed as diagnostic tools for the detection of pathogenic species.

Non-molecular techniques can be useful despite their limitations in sensitivity and specificity, particularly for anaerobic species, as well as for discovery and scaling. They can improve the robustness of results via identification of specific species of interest or identification of unidentified sequences that may belong to a known organism [101], when used in combination with molecular techniques, such as 16S rRNA high throughputs sequencing. Other major benefits of non-molecular techniques include that the materials are inexpensive and that the protocol requires limited equipment. However, specific culturing conditions that select for specific microbes, of which there must be prior knowledge, mean that many species can go undetected [102, 103]. Moreover, they are time consuming and labour intensive when performed at large scale. Thus, traditional culture techniques have largely been displaced by molecular techniques due to these time and labour issues, as well as these older techniques’ restricted insight into microbial communities.

### Molecular Based Approaches

Molecular techniques, including 16S rRNA high throughput sequencing, fingerprinting, microarrays and quantitative PCRs, are rapid, sensitive and highly specific, particularly for commensal organisms. Molecular techniques have rapidly replaced non-molecular techniques for use in identifying microbiome composition since their advent, due to these benefits (Fig. 2). The utilisation of genetic information to differentiate between taxa has made a more detailed exploration possible, and may provide information on the abundance and composition of these microbial communities beyond those routinely grown in the laboratory. The most described microbiota include bacterial communities, which can be identified through utilisation of the variable regions of the 16S ribosomal RNA (16S rRNA) gene, which is flanked by highly conserved regions. DNA is extracted from faeces in this method, commonly using a commercially derived extraction kit. It is amplified by PCR and then differentiated into groups based on similarity to identify the taxa present, allowing deep community sampling. Samples must first be collected and stored and the DNA extracted for all 16S and other molecular techniques.

### Sample Collection

Sample collection protocols will vary depending on study design. However, the timing of collection is an important factor to consider when comparing results across studies or during study design. Most studies provide specific time points, based on the gestational or post-gestational age of the infants. However, there are studies that group samples together more broadly, for example, binning samples together as “early-infancy” or meconium. Meconium is the earliest stool of a mammalian infant, comprised of a thick tar-like substance that lines the intestine of the unborn infant. Typically, meconium is not released until after birth. However, sometimes it will be released into the amniotic fluid prior to birth. It can also be released at different time points post-delivery, typically within the first three to five days. It may be that accurate comparisons cannot be made across studies when using such broad definitions, as the infant microbiome is dynamic, with choregraphed abrupt changes in composition [34, 104]. Therefore, the timing of collection is an important factor to take into consideration when interpretating the literature and planning future study design.

### Impact of Storage Conditions and Extraction Protocol on DNA for Molecular Techniques

#### Sample Storage

Storage conditions influence the stability and constitution of faecal microbial communities [105], which could prejudice study conclusions. Inadequate storage can lead to continued growth of specific organisms, altering the proportions of taxa/genera in a sample, and can lead to DNA/RNA fragmentation. Studies show DNA/RNA fragmentation can occur after 24 hours when samples are stored at room temperature [105], and that significant changes in bacterial communities can occur in samples after this time [105–107]. Potentially, changes can occur in as little as 30 minutes, as demonstrated by Gorzalek, et al. [108]. Currently, available storage methods include freezing or refrigeration at different temperatures, and the use of anaerobic incubation systems, aqueous storage/transport mediums and faecal occult blood tests.

#### Freezing and refrigeration

Optimal sample storage conditions depend upon the duration of storage. If samples are to be processed immediately, storage on ice for up to 48 hours [109], or 4°C for 24 hours [110] appear to be sufficient for sample preservation. However, immediate freezing of faecal samples to inhibit bacterial growth is the optimal procedure for longer term storage. Long term storage of faecal samples at -80°C has been shown to yield microbiota similar to that of fresh samples [106, 111, 112]. Storage of faecal samples at -20°C also has shown similar efficacy in sample preservation across several studies [105, 113, 114]. However, this appears to be time limited, with some studies reporting changes in taxa over longer-term storage at -20°C for storage times greater than a week, resulting in significant changes to *Bacteroides* spp. [115] and for up 53 days in the Firmicutes to Bacteroidetes ratio [116]. Storage at -80°C produces the most consistent results and appears to be the most common preservation method (Fig. 2). It was common for samples to be stored at -20°C or 4°C temporarily, or to use non-freeze methods until storage at lower temperatures was possible in situations where immediate freezing was not possible, such as with at home collection.

#### Other storage techniques

Immediate freezing of faecal samples can be logistically difficult, especially for large-scale population-based studies, and freeze-thawing effects may significantly diminish sample integrity. Therefore, other preservation methods may be better suited for some protocols. These preservation techniques include chemical and drying preservation such as DNA/RNA Shield and anaerobic incubation systems. Preservation buffers, aqueous reagents that stabilise and protect cellular DNA/RNA, like DNA/RNA Shield (Zymo Research) and RNAlater (Thermofisher), may also preserve genetic integrity for weeks without refrigeration or freezing [117–123]. Using these buffers ensures that samples are also protected from the potential stress caused by freeze-thawing effects. These buffers and other non-freeze preservation methods are a good alternative when freezing is not feasible.

Some potential issues have been highlighted with non-freezing preservation methods, despite these methods being a more practical alternative. Preservation buffers may result in lower diversity [124, 125] relative to immediate freezing. Moreover, some older preservation buffers may impede downstream DNA extraction and amplification of target genes [117]. Anaerobic incubation systems, like Anaerocult®, are only effective for storage of anaerobic strains and thus have obvious limitations. Most current research still supports the efficacy of preservation buffers, despite several studies highlighting their limitations. Thus, both freezing at -80°C and suspension in a stabilisation buffer are acceptable practices when considering all the available storage options.

#### DNA Extraction

The first step for molecular analysis is DNA extraction, which can be carried out using commercially available kits. Extraction is an important step in molecular techniques, which involves separating DNA from the other cellular material contained within samples of interest. The process involves cell lysis, or the disruption of cell walls, separation of the DNA from the other cell components and its subsequent isolation. DNA extraction can be laborious and carries a high risk of sample contamination, as a significant amount of handling of the biological material is involved. Fortunately, there are several commercially available extraction kits that make the process less laborious, more streamlined and more reproducible due to the widespread interest in the human microbiome.

The amount of tissue needed for DNA extraction and sequencing is dependent on the extraction methods and downstream application respectively. Generally, 100 to 1000 nanograms of DNA is required for whole genome sequencing, and as little as 1 to 10 nanograms for amplicon sequencing [126]. The amount of stool required will be dependent on the efficacy of the protocol in extracting the DNA, which is dependent on the methods used for the given protocol. The QIAamp DNA Stool Kit, the most commonly used in the preterm infant microbiome field, is optimised for 190-220 milligrams of stool, but as mentioned above, the amount of stool required is kit-dependent.

Unfortunately, different extraction protocols and kits can contribute greatly to variation in microbial community structure [127, 128], introducing bias to outcomes [115, 129, 130]. In one study the extraction method was demonstrated to be the second-greatest contributing factor to variation [127]. This variation arises in large part due to different methods of homogenisation and lysis. These steps are critical, as different stool fractions can contain different microbial compositions, and different microbes are lysed better by different techniques.

Microbial cell wall structure differs between Gram-negative and Gram-positive bacteria and require different lysis methods for DNA extraction. In Gram-negative bacteria, the cell wall is thin and made up of both a peptidoglycan and phospholipid bilayer containing lipopolysaccharides, whereas Gram-positive bacteria have a thick peptidoglycan cell wall. As a result, Gram-positive microbes require more vigorous lysis methods and Gram-negative microbes are more easily lysed [115].

Different forms of homogenisation or lysis can, therefore, contribute to bias by not effectively disrupting the cell wall of all microbes present in a sample or, conversely, by destroying the DNA of easily lysed cells. Mechanical, chemical, and enzymatic lysis methods can also produce different proportions of taxa, with mechanical methods producing higher bacterial numbers and greater diversity [115]. Two comprehensive studies that explored several kits, including the QIAamp DNA Stool Kit, found that the International Human Microbiota Standards (IHMS) Protocol Q, that includes mechanical lysis, performs best across several parameters [128, 131]. Despite this, our review found that the most common extraction method utilised in premature infant microbiome studies was the Qiagen QIAamp DNA Stool Kit. This method uses a combination of heat, chemical and enzymatic lysis, with some studies adapting the protocol to add mechanical lysis through bead beating. Unfortunately, different bead-beating instruments have been shown to produce bias [128, 132, 133]. Despite this, mechanical disruption is essential for comprehensive profiling of the human gut microbiome [133, 134], and until a standardised protocol is established, researchers must be careful to consider the bias generated through different kits.

### Molecular Techniques

#### **Fingerprinting** Methods

The increasing usage of 16S rRNA amplicon sequencing has been matched with a reduction in the use of other techniques, like different fingerprinting methods. Fingerprinting methods are more cost effective and faster to perform [135], although high throughput sequencing techniques provide a broad detailed analysis of microbial communities. These techniques are favoured in comparison to traditional culture methods, as they provide greater sensitivity and specificity for individual organisms, and can be used to analyse large numbers of samples [135]. Broadly speaking, fingerprinting methods provide a profile of microbial communities that uses amplification of a target gene (commonly the 16S rRNA gene) and the utilisation of gel electrophoresis to observe physical separation of amplicons, allowing exploration of highly abundant taxa. Fingerprinting methods have been used in studies exploring the preterm microbiome (18.5%), although they are currently less common. These techniques include denaturing/temperature gradient gel electrophoresis (D/TGGE) and terminal restriction fragment length polymorphism (TRFLP), as well as denaturing high performance liquid chromatography (dHPLC) in a single study. dHPLC uses liquid chromatography to identify polymorphisms [136], while the others rely on electrophoresis to differentiate between sequences, although all are considered fingerprinting methods.

#### Denaturing/temperature gradient gel electrophoresis

Gradient electrophoresis is the size dependent movement and separation of dispersed nucleic acids through an acrylamide gel. As DNA has a negative charge, it moves through the acrylamide gel or molecular mesh towards the positive electrodes at a rate that is inversely proportional to the size of the nucleic acid sequences, thus allowing the differentiation of different sized sequences [137]. More detailed exploration is achieved by applying either a temperature (TGGE) or chemical gradient (DGGE) to denature the samples as they move across acrylamide gel, based on the chemical make-up of the sequences [138, 139].

Both DGGE and TGGE differ in the mechanism of DNA denaturation. In DGGE the nucleic acids are exposed to increasingly extreme chemical conditions, leading to the denaturation of the DNA in a stepwise process. This allows for visualisation of the sequence differences by their position on the gel. The method relies on differences in the ability to denature the bases, which is determined by base pair sequences to separate genes by size. In contrast, TGGE uses a temperature gradient in combination with the electrophoresis. Strands separate across the gel depending on base-pair content, with smaller molecules travelling faster [139] as the temperature increases.

#### Terminal restriction fragment length polymorphism (TRFLP)

TRFLP also uses electrophoresis to differentiate between sequences based on terminal restriction fragment size, like TGGE and DGGE. This allows sequence identification for microbial community profiling [140]. The method involves PCR amplification of a target gene with fluorescently labelled primers and subsequent digestion with restriction enzymes. The sizes of the different terminal fragments are then determined by separating the fluorescently tagged terminal fragments via capillary or polyacrylamide electrophoresis in a sequencing gel, creating unique banding patterns allowing identification of microorganisms [141]. T-RFLP has high throughput capability and can be highly sensitive, but it also has limited accuracy as incomplete or non-specific digestion can lead to overestimation of diversity. Homology of sequences can contribute to an underestimation of taxa present [142]. Furthermore, libraries must be built prior to analysis.

#### dHPLC

dHPLC uses liquid chromatography to identify DNA polymorphisms [143], unlike TGGE, DGGE and T-RFLP. DNA strands are separated into hetero- and homoduplexes using an ion-pair, reverse-phase liquid chromatography on a poly alkyl column matrix [143]., following partial heat denaturation. The presence of polymorphisms is revealed by the differential retention of these homo- and heteroduplex DNA fragments [144]. Heteroduplexes are double stranded DNA that have formed during PCR amplification that are mismatched at the site of mutation. Mismatched double stranded DNA fragments have reduced retention on the column matrix, and subsequently in a reduced retention time, thus allowing for identification of polymorphisms. As a result, dHPLC can be useful as a screening test for mutations that may be involved in diseases or associated with antibiotic resistance [136, 145]. The method has also been used to differentiate between taxa at species depth by applying the same underlying principle of scanning for mutations to the detection of sequence variations between PCR-amplified bacterial 16S rRNA genes [146], and also as a tool for re-sequencing of genomes [144].

#### Limitations (Pros and Cons)

Some consider dHPLC to be the optimal fingerprinting method [70], potentially allowing identification of bacteria at the species and/or biotype levels [146]. However, these techniques require extensive downstream processing, can produce PCR bias [102, 147] and have limited detection depth, as it is difficult to relate banding patterns created in gels to species or lineages created by fingerprinting methods [135]. Thus, fingerprinting methods are usually limited to identification at the order/family level [148], and to only the most abundant organisms. This methodology also makes it difficult to combine data from multiple studies into a single analysis [135]. Fingerprinting techniques can be useful for exploring dominant members of microbial communities, including clustering of communities based on dominant members [149]. However, their application is limited in describing entire microbial communities.

#### Phylogenetic Microarrays

Microarrays were originally developed to monitor gene expression, but their application has been expanded to include comparative genomics, DNA sequencing analysis, single-nucleotide polymorphism (SNP) analysis and microbial detection [150], including studies on the premature infant microbiome [77, 78]. Microarrays are microscopic slides printed with probes made of predefined oligonucleotide sequences complementary to the small subunit (SSU) rRNA. The oligonucleotide probes detect gene expression or mRNA transcripts expressed by specific genes and extracted from target organisms. Reverse transcriptase converts mRNA into complementary DNA (cDNA), and this cDNA is fragmented and fluorescently labelled and added to the microarray [151]. cDNA then binds complementary oligonucleotide probes via hybridisation, and measurement of the observed fluorescent intensity at a given probe is an indication of the abundance of predetermined sequences that are chosen prior to analysis and are of interest [151]. This makes phylogenetic oligonucleotide arrays (phyloarrays), including HITChip, suited to the analysis of microbial communities.

HITChip is an ecosystem-specific phylogenetic microarray developed for microbial detection in the human gastrointestinal tract [152, 153], and is the only microarray to be used in studies on the premature infant gut microbiota. HITChip is an oligonucleotide microarray that uses 4,800 oligonucleotide probes based on two hypervariable regions (V1 and V6) of the 16S rRNA gene, identifying 1,140 phylotypes. Phylotypes were designed following the analysis of 16,000 human gastrointestinal tract 16S rRNA gene sequences [153]. As a result, HITChip is highly specific to the human gastro-intestinal tract microbiome and provides a high level of diversity.

#### Limitations (Pros and Cons)

Benefits of microarrays include ease of use, speed and cost [154], and potential for investigating microbial gene functionality [155]. These intermediate methodologies allow processing of large sample sizes, while providing more taxonomic depth, like fingerprinting methods. Microarrays target the ribosomal RNA gene, allowing comparisons of diversity and taxonomy, and thus display similar robustness [156, 157], like 16S rRNA sequencing. However, when compared to high throughput techniques, phylogenetic arrays are limited when assessing new lineages, as they can only detect predefined taxa [135, 158]. Other methodologies are better suited when there is potential for taxonomic or gene discovery, as microarrays are limited to predefined taxa. Microarrays are not commonly used in studies on the premature infant gut microbiome, as it is a relatively new area of study, and other methodologies may be better suited for characterising this niche.

#### qPCR and Fluorescent In-situ hybridization

Polymerase chain reaction (PCR) is a highly sensitive molecular technique that was originally developed for detection of DNA/RNA sequences, but has since progressed beyond purely nucleic acid detection. Quantitative polymerase chain reaction (qPCR) builds on standard PCR by providing the quantity of amplified genes. qPCR also differs from standard methods as it monitors the amplification of targeted DNA molecules in real time or during PCR instead of at the end. This process allows not only detection, but also quantification and characterisation of nucleic acids [159]. Fluorescent dye is added to the PCR reaction in dye-based qPCR, and the fluorescent signal increases proportionately to the quantity of DNA being replicated. This allows quantification of DNA after each cycle. However, qPCR only allows one target to be examined at a time, thus throughput is limited. The more accurate probe-based qPCR provides one way around this drawback, by simultaneously examining multiple targets via recognition of sequence-specific probes. The fluorescent signal from the probe in probe-based qPCR is proportional to the target sequence that is present in the reaction [160], as it is in dye-based qPCR.

Fluorescent in situ hybridisation (FISH) is another probe-based technique. FISH is a molecular technique that uses complimentary binding to identify or quantify cDNA that can be used for microbial identification, like microarrays and qPCR [161]. FISH uses fluorescently labelled DNA probes that match specific DNA sequences that can be observed under a microscope, allowing direct quantification of specific taxa. Fluorescent oligonucleotide probes are created for targets, either 16S or 23S rRNA sequences. The target and probe sequences are denatured with heat or chemicals, and mixed together prior to hybridisation. Hybridisation then occurs between complementary target and probe sequences, with fluorescence microscopy facilitating detection of hybridisation via observations of fluorescently labelled cDNA. This target-specific methodology facilitates high accuracy when targeting specific microbes.

#### Limitations (Pros and Cons)

Both qPCR and in-situ hybridisation can provide highly accurate quantification [162], can be highly sensitive [163], and can produce similar results to metagenomic methods when considering the main intestinal microbial groups [24]. However, they are limited in their application, as prior knowledge of sequences is required, like fingerprinting and microarrays. Thus, these methods have no discovery power and no capacity for assessing diversity. FISH has been designed to examine the major microbial groups present in premature infants, but is based on groups present in full-term infants [79, 80]. However, predefining taxa in this way is a significant limitation, as premature infants are known to have significantly different microbial populations to infants born full-term [24, 26]. Moreover, both qPCR and FISH are not scalable, and therefore are only effective for low target numbers. qPCR or in-situ hybridisation methods may be beneficial when specific populations are being targeted, as they have limited bias and are cheaper compared to sequencing methods, but they are not suitable for projects mapping entire microbial ecosystems, like that of the premature infant gut microbiome.

### Sequencing Techniques

DNA sequencing is the process of nucleic acid sequence determination, and covers a broad range of techniques across three generations of sequencing. The first generation of sequencing began with a low throughput technique, Sanger sequencing, which only sequenced a single DNA fragment at a time [164]. Sanger uses a labour-intensive cell-based amplification step, involving cloned sequences being placed into plasmids for amplification, prior to extraction and purification [164]. The second generation of sequencing techniques, often referred to as next-generation sequencing (NGS) or high-throughput sequencing (HTS), involved 16S rRNA Metabarcoding and Metagenomics (shotgun sequencing). NGS refers to any sequencing method using the concept of parallel processing. This parallel processing increased the volume of reads per run to millions, vastly improving efficiency, as did the development of a cell-free system. NGS also runs elongation and detection steps in parallel, again improving efficiency [165]. However, NGS technologies are limited in that they use short reads (bp), which create a computational challenge when assembling or mapping to genomes. A third generation of sequencing was developed to overcome this challenge, long read or single molecule direct sequencing. The capacity of all sequencing technologies to produce large volumes of relatively accurate data, coupled with the continual reduction in cost, has led to their adoption across most modern studies investigating microbial populations. 16S rRNA high-throughput amplicon sequencing (metabarcoding) is now the most common method used for studies specifically characterising the preterm infant’s gut microbiome (Fig. 2). However, other methods, including shotgun metagenomics, and third generation single molecule direct (long-read sequencing), have also been applied. All techniques described have their strengths and limitations.

### Next Generation Sequencing

#### 16S rRNA amplicon sequencing

16S rRNA amplicon sequencing, or metabarcoding, has become the most common technique for characterisation of the preterm infant microbiome since it was first used in 2004. 16S rRNA metabarcoding uses high throughput sequencing to target variable regions of the 16S rRNA gene, allowing accurate identification of microbial community composition [166–168]. The 16S rRNA gene codes for 16S ribosomal RNA, a component of the 30S small subunit of prokaryotic ribosomes. The 16S rRNA gene is highly conserved across taxa, but also has several variable regions allowing differentiation between taxa, due to a slow rate of evolution. The variable regions are conserved enough that most taxa can be characterised, but variable enough that taxa can be differentiated. There are nine of these hypervariable regions that range in base pair length and are involved in the secondary structure of the small ribosomal subunit. The regions vary in conservation, and thus different regions correlate with different levels of taxonomic resolution. The protocol for 16S rRNA gene amplicon sequencing involves DNA extraction, PCR amplification of the variable target region(s), grouping of sequences into OTUs, ASVs or an equivalent, and then mapping these sequence variants to a reference database for taxonomic identification.

16S rRNA metabarcoding is the most common technique for characterising the preterm infant. However, despite this there is no predominant protocol. There are a myriad of options at all stages of the workflow, all of which can introduce bias that alters outputs, which is supported by the observation that samples cluster by study [15]. Technical differences in how samples are collected and stored, how DNA is extracted, the primers that are selected and variable regions targeted, the sequencing platform, bioinformatic pipelines and reference databases could all produce systemic bias that obscures biological differences [15, 16]. As the 16S rRNA protocol is the most common technique for characterising the preterm infant gut microbiome, a more detailed explanation of its varied protocols is discussed below. Caution should be used during both interpretation of the literature and study design until a standardised protocol is agreed upon.

#### Selection of Variable Regions and Primer Bias

Once DNA is extracted, and prior to sequencing, the target DNA from the variable region of interest must be amplified via PCR. However, there is much debate on which variable sub-region to target and matching primers to use. Indexing primers are complementary base pair sequences that are required to ‘select’ and amplify variable sub-regions. These 16S rRNA variable sub-regions can vary by up to 40% in taxa between samples analysed with the same pipeline [169], and it has been argued that the most critical step for accurate amplicon analysis is the choice of primers [170], as primer selection can alter coverage [171].

Samples with the same extraction and storage protocols have been demonstrated to cluster by primer selection [15]. This is because poor primer selection can influence quantitative abundances [172], and contribute to under-representation or over-representation of taxa [173, 174] or selection against particular taxa [135, 175, 176]. For example, most primers may inadequately detect Bifidobacterium [177], possibly over-exaggerating the low levels already observed in preterm infants. For identifying species, targeting of these hypervariable sub-regions is limiting, as different sub-regions show bias in the taxa that they can identify due to limited variability within the sub-region itself. Thus, while V1-V3 may be good for *Escherichia* and *Shigella* species, *Klebsiella* will require V3-V5, and *Clostridium* and *Staphylococcus* require V6-V9 sequencing [178]. As a result, studies that target hypervariable sub-regions must settle for taxonomic resolution at the genus level. Thus, the only way to ensure good taxonomic identification would be to sequence the entire 16S gene, given these limitations and the bias that can be introduced through variable region and primer selection. However, high error rates and cost are still major deterrents.

Arguments have been made for targeting the V4-V6 [179], V4 [172, 179, 180], and V3-V4 [169, 171, 181] sub-regions, with V4 being the most common for characterising the microbiome of preterm infants, when targeting hypervariable sub-regions for high throughput sequencing (Fig. 2). The Earth Microbiome Project [182] recommends the V4 sub-region, and it has been demonstrated to have low PCR and sequencing errors due to complete overlap of paired end sequences [180]. Other work has also shown that the phylogenetic relationships based on V4 were closest to that entire 16S rRNA gene [179]. However, some evidence suggests that targeting the V4 region may not be as accurate as previously thought [178]. The debate about which region is best is ongoing, but research conducted by Almeida et al. makes a convincing argument for the use of the V3-V4 region above all else. It compared variable regions across different combinations of pipelines and reference databases for both mock communities and simulations [169], and found the V3-V4 region consistently produced the most reliable taxonomic inferences. Taken together, the frequent use of V4 and the findings across studies, targeting either the V4 or V3-V4 sub regions may be best practice until standardisation occurs. Additionally, consideration should be given when making comparisons across studies that use different variable regions.

#### NGS Platform

Several sequencing platforms are available for 16S rRNA short read amplicon sequencing, with Illumina MiSeq, Roche 454 (originally 454 Life Sciences) and Thermo Fisher’s Ion Torrent Personal Genome Machine (PGM) all being used in the context of the premature infant microbiome. Roche 454 has historically been the dominant platform, as NGS technologies began with it. However, Illumina now dominates the market, with its consistent growth and the eventual abandonment of the Roche 454 sequencing platform in 2016. It is important to understand the differing methods across platforms, and their limitations and biases for accurate interpretation of the literature, although most modern sequencing technologies will opt for Illumina sequencing.

Illumina sequencing technology facilitates massively parallel sequencing by using optical signals to detect base pairs in real time. DNA libraries, containing fragments that vary between 100-150bp, are loaded onto a flow cell and placed in the sequencer for this process. The sequences bind to the flow cell via complementary adaptors. A process called clonal bridge amplification or cluster generation then amplifies each read, creating a spot (cluster) on the flow cell (slide) with thousands of copies of the same DNA strand. Then, through a process coined sequencing by synthesis, fluorescently tagged nucleotides bind to the complementary bases on the DNA strand via repeated cycles of single-base extension. A fluorescent signal (the colour of which is dependent on the base) is emitted upon incorporation of each nucleotide, and a picture taken, indicating what nucleotide was added. Once the forward DNA strand is read, the reads are washed away, and the process is repeated for the reverse strand. Computers then construct the sequence by detecting the base at each site in each image.

Roche 454 relies on the production of sequence clusters, like Illumina, but through a process called clonal emulsion PCR (emPCR). In emPCR, single stranded DNA fragments (up to 1kb) from a DNA library are attached to the surface of a bead, rather than a slide, with one bead for each DNA fragment. The reads bind to the bead via complementary adaptors. The beads are then compartmentalised into single wells containing emulsified oil, and are subjected to thermal cycling to achieve clonal amplification. This process produces many copies of the original template, as in Illumina’s clonal bridge amplification. The slide containing the wells is then flooded with one of the four nucleoside triphosphates (NTP) that bind to their complements, releasing a light signal upon addition. The original NTP mix is washed away and the next NTP is added and the cycle is repeated. The light intensities are then plotted on a graph for each sequence read, with graphs then used to determine the sequence computationally.

Ion Torrents PGM also uses clonal-emPCR, but differs from Roche 454 both in how it determines the nucleotide sequences and the size of DNA fragments. Ion Torrents PGM or proton sequencing uses DNA fragments of ~200bp, which are again bound to beads via adaptors. These then undergo PCR and are washed with different NTPs. It then exploits the release of hydrogen ions, which occurs through the addition of an NTP to a DNA polymer for nucleotide sequence determination. The release of hydrogen ions causes changes in pH that are used to determine the DNA sequences.

#### Limitations (Pros and Cons)

No platform is without its limitations: limitations that can contribute to platform-associated biases and study-based clustering [15], despite significant developments in the sequencing field. For example, Roche can have high sequencing error rates associated with A and T bases [183], high error rates in homopolymer regions resulting from accumulated variance in light intensity [184–186], and can have up to 15% of sequences resulting from artificial amplification [187]. Ion Torrent is also subject to high homopolymer error rates [186, 188, 189], as well as organism-specific read truncation, due to the similar methods of Roche and Ion Torrent, in which multiple nucleotides can be incorporated during a single cycle [190]. Illumina still have their own systematic base-calling biases [191], even though platforms produce comparatively lower error rates [186]. These include production of homopolymer-associated sequencing errors [183], different quality reads across different sequencing tiles [192], increased single-base errors associated with GGC motifs [193] and different sequencing error rates at the different read ends [194]. In the two dominant platforms in preterm infant studies Roche 454 and Illumina, the differences caused by platform are minor [172, 183]. However, the lower error rates, higher throughput [186] and higher read quality [190] achieved by Illumina, results in higher quality data. This allows stringent quality control parameters, resulting in more reliable outputs for downstream analyses [172].

#### Bioinformatics and Reference Databases

Bioinformatics is an interdisciplinary field of science that combines biology, computer science and statistics, in order to process large amounts of biological data, such as that produced by 16S rRNA gene amplicon sequencing. Bioinformatic tools like QIIME [195] and Mothur [97] are required to clean up and make inferences on microbial composition from data that is not human-readable post sequencing and prior to downstream analysis. Bioinformatic tools or pipelines need to be both precise and reliable in order to produce accurate biological conclusions using the vast amounts of genetic data that is being produced with sequencing. These tools convert raw data into interpretable taxonomic abundances by comparing sequencing reads in the form of OTUs, ASVs or an equivalent (sequence variants that represent a true sequence) [196] to a defined reference database, identifying the taxa present in samples by assigning the most likely taxonomic lineages. The accuracy of the taxonomy classifications produced is then reliant on both the diversity and breadth of annotated sequences in the reference databases [169], as well as the accuracy of the ever improving algorithms used by bioinformatic pipelines.

There is no agreement on optimal practices, although the bioinformatic pipelines are rapidly changing and improving, and many researchers are unaware of the biases associated with using different tools. Combinations of different software packages, databases and targeted regions can produce vastly different levels of accuracy when examining mock communities and running simulations [169]. When comparing several bioinformatic tools: QIIME, QIIME 2, Mothur and MAPseq [197], Almeida et al. found that QIIME 2 was the optimal tool in regards to detection sensitivity and composition prediction. QIIME 2 had the largest proportion of classified sequences at the most accurate relative abundances [169]. However, MAPseq was more precise, with fewer genera being miss-assigned. A more recent study which compared the most popular current bioinformatic pipelines for 16S rRNA gene amplicon sequencing, found that DADA2 was the best choice for studies requiring the highest possible biological resolution, but that USEARCH-UNOISE3 [198] had the best overall performance [199]. The common theme running through USEARCH-UNOISE3, DADA2 and QIIME 2 (uses DADA2/deblur plugins) is their denoising or clustering algorithm.

Denoising and clustering are methods for correcting sequencing errors through grouping of similar sequence variants into a bin. This was originally done through OTU clustering, in which sequences are clustered based on a 97% similarity threshold. However, there are several methods for implementing this threshold: closed-reference, open-reference and de novo clustering [200]. The de-novo method clusters reads against one another, based on the threshold, without a reference database, unlike the reference-based approaches. In contrast, the closed-reference method clusters reads against a database and excludes those sequences that do not align. Open-reference clustering also clusters against a database, but then clusters reads that do not align de novo. The most successful method is debatable [200, 201], but may be dependent on the study design. Nonetheless, the quest for more reliable data has seen a shift away from OTU-clustering towards error modelling, which takes into account both abundance and error.

Denoisers, as seen in DADA2 [202] and deblur [203], generate error models learnt from the reads and use these models for sequence variant assignment with either ASVs (DADA2) or subOTUs (deblur). The error modelling approach allows for clustering down to the level of single-nucleotide differences in the sequence region, improving resolution, and allows consistently reproducible labels with intrinsic biological meaning [196]. These improvements allow researchers to distinguish between true sequences and those generated during PCR amplification and sequences, and result in comparable general community structure across different tools. However, some variability still exists, with differences in the number sequence variant produced and resulting alpha diversity [204], despite these improvements. These differences should be considered when making cross-study comparisons and during study design.

The most common bioinformatic pipeline for studies exploring the preterm infant gut microbiome is QIIME. QIIME’s use of OTU clustering and its production of a large number of spurious OTUs and inflated alpha diversity [199] should be taken into account when considering older literature. However, QIIME was succeeded by QIIME2 in 2018 (first published in 2019) [96], which uses an updated error modelling approach, with either DADA2 or deblur plugins. Most studies on the preterm infant microbiome predate the release of QIIME2, and therefore will have used the older version. It is unclear if newer studies will make the transition, due to the limited number of papers released since the pipeline’s publication. However, in order to produce more robust data, research in this field needs to move towards these new and improved methods.

Along with choosing the best bioinformatics tool, the reference database used is also an important consideration. Pipelines can use a homology based or Bayesian approach to match sequence variants to sequences from the reference databases. This was originally achieved with a similarity threshold of >95% sequence match being considered to represent the same genus and >97% match for species level identification [205]. However, recent work suggests that these thresholds are too low for accurate assignment [206]. The reference databases contain FASTA files with reference sequences assigned to nodes of taxonomy. However, discrepancies exist in both nomenclature and lineages of taxonomy between databases [206, 207], which has obvious implications for the taxa identified in a sample. The Ribosomal Database Project (RDP) is the database used most often for studies on the preterm infant microbiome. SILVA may have better recall and be more precise than the more commonly used RDP (Fig. 2), when examining the human microbiome, based on a benchmarking paper by Almeida et al. [169]. However, more research on best practices, along with standardisation across databases is needed.

#### Bioinformatics – Contamination

Contamination is another way variability between studies can be introduced. DNA contamination from collection, extraction and sequencing protocols (and kits) can impact upon the interpretation of results [208, 209]. Many of these contaminants could be considered normal inhabitants of the human gastro-intestinal tract, and so it is important that measures are taken to mitigate the risk of contamination, and that steps are taken to account for or remove this contamination. Negative controls, spike-in controls and microbial standards, when coupled with appropriate bioinformatic tools, are effective ways to account for both accuracy of techniques and contamination. Bioinformatic tools like microdecon [210] can be used to remove homogenous contamination. This is important for both study design and interpretation of the literature, as contamination may produce unusual or novel findings if the appropriate mitigation strategies are not in place [208].

#### Descriptive metrics

Analysing microbial data can provide a significant challenge due to the volume and complexity of the data. Additionally, it is difficult to provide a best practice approach for statistical analysis of microbiome data, as it is highly dependent on the hypotheses and objectives of the study. However, generally, there are three main metrics that are considered in microbial analysis: alpha diversity, beta diversity and differential abundance. Unfortunately, finding a meaningful way to conduct these analyses can be a convoluted process.

Alpha diversity refers to the diversity within a sample, summarising the ecological structure with respect to either richness (number of taxonomic groups), evenness (distribution of abundances of the groups), or a combination of the two [211]. Alpha diversity can be represented by richness, the Chao 1 index, the Shannon-Weiner index, the Simpson index, Pielou’s evenness or Faith’s phylogenetic diversity. All these indices differ in what they represent, with richness and the Shannon-Weiner index common in the context of microbiome research. Richness is simply referring to the count of sequence variants, whereas the Shannon-Weiner index takes both richness and evenness into account. The Chao 1 index is a bias-corrected richness estimate [212] that has become less common with the advent of newer pipelines (e.g. DADA2), due to their handling of singletons, which is based on the assumption that many are spurious sequencing variants.

Beta diversity refers to microbial differences between samples or groups. There are several metrics for beta diversity that represent distances between samples, as there are in alpha diversity. Beta diversity metrics include the Jaccard distance, Bray-Curtis distances and UniFrac. Jaccard distance is the number of sequence variants shared by samples divided by the number shared. Bray-Curtis distances, one of the most widely used in microbiome research, builds on this by taking abundances into account as well, whereas UniFrac distances, either weighted or unweighted, represent the differences between samples based on phylogenetic differences. These distance matrices can all be represented with direct comparisons of distances between samples or groups, hierarchical clustering or ordination techniques. Ordination is the most common as it reduces the complex distance data to a 2D or 3D plot, making for easy interpretation.

Alpha and beta diversity, along with differential abundance, all require normalisation prior to analysis. Differential abundance is simply making comparisons for taxonomic abundance between samples or metadata. However, the process is more complicated than simply counting the number of reads per sample. This is because using read counts as a measure of abundance is flawed, as the number of reads is actually an artefact of sequencing, and is therefore not a good representation of abundance [213]. Alpha and beta diversity also take read counts into account, and so both are also sensitive to sequencing depth. Both diversity metrics require an equal number of reads per sample for valid analysis. This means that if appropriate measures are not taken, library sizes can determine diversity results [214]. Thus, reads must be normalized to account for the differing number of reads per a sample prior to analysis. However, although normalisation is the solution, different methods are required for different analyses.

Older methods of normalisation include Total Sum Scaling/Normalisation (TSS) and rarefying. In TSS, data is transformed to proportions by dividing the reads for each sequence variant by the total number of reads, whereas rarefying adjusts for differences in library sizes by assigning a sequencing depth threshold, and subsequently subsampling samples with a depth above the threshold and discarding those below. However, both methods are poor options for differential abundance testing and can have high type 1 errors [215–218]. Additionally, TSS doesn’t account for heteroskedasticity [17] and rarefying discards potentially useful data. As a result, other modern methods have begun to replace the old methods.

Newer methods include variance stabilizing transformation with DESeq2 [219], upper quantile normalisation [215], CSS normalisation [220] and Trimmed Means of M-values (TMM) with EdgeR [221]. Methods like DESeq2 and EdgeR are generally favoured due to their performance across several comparative papers [215–217]. However, these comparisons are specific to standardisation of within-sample variance, the ability of data to cluster in ordinations, and their performance in differential abundance testing, but there are several important limitations [222]. These methods tend to focus on standardising within-sample variance across samples, as they were created for differential abundance testing. As a result, the newer methods do not guarantee equal number of reads across samples. They supress species evenness and overestimate the importance of low abundance taxa (through log transformations) [222]. The overestimation of low abundance taxa and suppression of evenness can contribute to an inaccurate representation of the community, and, along with non-equal read counts, can lead to inaccurate comparisons between samples.

So, although proportions, specifically TSS and rarefying, are not suitable for differential abundance testing, they are more suitable for diversity analysis, as they give a more accurate representation of the microbial communities while accounting for differences in read depths [218, 222]. Additionally, methods like the variance stabilising transformation with DESeq2 are favoured for differential abundance testing. It is critical that researchers are aware of the strengths and limitations of normalisation methods for accurate interpretation and robust study design and differences between the subsequent analyses.

#### Metagenomic Shotgun Sequencing (whole genome sequencing)

Shotgun metagenomics is another NGS approach that has been used a handful of times for characterising the gut microbiome of premature infants. Shotgun metagenomic sequencing targets all DNA in a sample, in contrast to 16S rRNA sequencing, which targets a specific region/gene. The protocols differ from 16S rRNA sequencing slightly, although they use the same sequencing technology. Shotgun metagenomics does not require amplification, as there is no target region/gene, but it does require the removal of host DNA prior to mapping, as all extracted DNA in a sample is sequenced. This alternative NGS method provides greater taxonomic resolution and gene annotation, allowing more comprehensive analyses. As a result, studies using this technology are typically looking to link functional gene profiles or pathogen strains to disease.

#### Limitations (Pros and Cons)

Shotgun sequencing has its limitations, despite its obvious benefits. There are numerous experimental and computational approaches that can be carried out at each step, as in 16S rRNA sequencing [223]. DNA extraction methods have been shown to affect composition [224], due to kits and reagents containing microbes [225] and differences in lysis techniques [226]. Library preparation and sequencing can introduce errors through PCR amplification [223] and selection of platforms [227, 228]. Furthermore, specifically metagenome profiling can cause protocol-associated variability, having several options for bioinformatics, as all metagenomic profiling techniques have their own limitations [223].

There are two approaches for metagenome-profiling: assembly-free methods and assembly-based methods. Assembly-free methods, also known as read-based profiling or ‘mapping’, make comparisons to reference databases that contain whole genomes, such as Kraken [229] or Centrifuge [230], or to selected marker genes, such as mOTU [231]. Alternatively, assembly-based analysis uses assemblers like Meta-IDBA [232] and SOAPdenonovo2 [233] to reconstruct genomes de novo. Assembly-based methods can construct multiple whole genomes and resolve novel organisms, but can be a significant computational burden and are limited in assessing complex communities. Alternatively, read-based analysis is computationally efficient and can deal with more complex communities, assuming there are enough sequencing depth and genomes in the reference database. However, identification is limited to those microbes previously defined, and so community structure/function is limited. Both approaches have their strengths and weaknesses, and which is best may depend on the question being asked.

There are also pros and cons when comparing NGS shotgun approaches to other sequencing methods. Metagenomics has more reliable species identification and broader analyses potential relative to metabarcoding, but the bioinformatics is more involved, requiring more time, skill and computational power, and the sequencing is more expensive, as entire genomes are being sequenced instead of a single gene. As a result, older studies using shotgun approaches tend to have lower sample sizes [2] or only use the technique on a subset of the cohort [74]. Additionally, where fragments of bacterial genomes are mixed in with contamination from host species and other organisms, 16S rRNA amplicon sequencing has specificity for bacteria, does not require full reference genomes, and does not require large quantities of, nor high quality, DNA [234, 235], as opposed to shotgun sequencing. However, the adoption of this technique will likely become more widespread as the price of shotgun metagenomic sequencing continues to drop, in combination with improved computational methods. However, 16S rRNA amplicon sequencing targeting variable regions continues to dominate studies in this field at the present time.

#### Third Generation Sequencing

Long read sequencing is another sequencing approach. Full length sequencing of the 16S gene was made possible with the advent of third generation sequencing technology, also known as long-read sequencing. This approach is possible with platforms like Oxford Nanopore Technologies Minion [236] and techniques like Pac Bio’s Circular Consensus and Continuous Long Read Sequencing [237]. These technologies allow discrimination between millions of reads that may only differ by a single nucleotide [178], and have the capacity to produce reads in excess of 10,000 base pairs (bp) [238, 239]. This allows the sequencing of the entire 1,500 bp 16S gene and increases the resolution in taxonomic profiling to species and strain level.

Third generation sequencing can produce these long reads because their design is distinct from previous sequencing methods. Nanopore technology produces long sequences by passing a single DNA molecule through a DNA pore, measuring changes in current across a membrane. The current passing through the membrane is dictated by the size of the base pairs in the sequence that is passed through the pore. Alternatively, PacBio’s SMRT (Single Molecule, Real-Time) sequencing repeatedly passes a DNA molecule through a DNA polymerase attached to a well, with short sequences being read until there are enough overlapping reads to identify the entire sequence.

Long-read technologies can also be applied to whole genome sequencing (WGS) and shotgun metagenomics, as well as full length sequencing of the 16S rRNA gene. WGS or shotgun metagenomic approaches allow greater sequencing depth, meaning species level detection of the preterm microbiome can be achieved, like sequencing the entire 16S gene. This capacity was demonstrated by Legget et al., who took advantage of Oxford Nanopore’s ability to produce near-real time data in developing a metagenomic screening platform for preterm infant microbiome samples [94]. Moreover, as shotgun metagenomic sequencing targets all genomic DNA in a sample, the data can be used for other analyses, like functional profiling and antibiotic resistance gene profiling. This provides a comprehensive investigation of microbial ecology.

#### Limitations (Pros and Cons)

The long reads produced from the two technologies are their major advantages. Nanopore can produce reads generally ranging from 10Kbp to 1Mbp, with the longest sequence produced being >2Mbp [240]. These longer reads, along with advances in the associated computational methods, allow for greater sequencing depth than short-read technologies, with potential for greater accuracy [241], as they can distinguish between sequencing artifacts and actual biological sequences [178]. However, high error rates [242–244] are still a problem in TGS, despite the claim of high accuracy. For Oxford Nanopore, this high error rate comes from using changes in current to identify base pairs [245]. In PacBio’s Single Molecule, Real-Time sequencing no current technology can precisely capture the rate of information produced (DNA polymerase adds 100bp/s), , which is one reason why the DNA must be passed through the enzyme multiple times to overcome this issue. So, although these technologies show promise, the high error rates and cost are still deterrents, which is probably why they have been used so seldom in studies on the preterm infant microbiome.

## Conclusions

Variability of results will continue to be a limitation when investigating microbial populations in premature infants until there is standardisation of protocols. This review aimed to describe the most common techniques used in researching the preterm infant microbiome, and their limitations. The objective was to provide those entering the field with a broad understanding, so that considerations can be taken for both literature interpretation and future study design. 16S rRNA amplicon sequencing is the most commonly used method, as it is cheaper than both long-read and shotgun metagenomic sequencing, more detailed than non-molecular techniques and allows the characterisation of taxa present across a wide range of samples. This approach, however, has several limitations that can introduce bias. Full length sequencing of the 16S gene or a shotgun metagenomics approach may provide better options, especially as accuracy continues to increase, along with a reduction in cost. However, until these options become more viable, 16S high throughput sequencing targeting a select number of hyper variable sub-regions will continue to dominate.

There are a number of options at different stages within 16S sequencing methods that can contribute to bias, and with the large number of tools and databases available, it can be a difficult task deciding on an optimal approach. In this work we briefly described the bias across methodologies, with emphasis on 16S techniques. The most commonly used techniques within 16S rRNA high throughput sequencing are sample storage at -80°C, QIAamp DNA Stool Kit for extraction, sequencing on the Roche 454 platform, targeting the V4 region, and using the QIIME or QIIME2 pipeline in combination with the Ribosomal Database Project reference database. However, the optimal combination for 16SrRNA sequencing would likely be storage at -80°C, an extraction kit that includes mechanical lysis, such as (IHMS) Protocol Q or the Power Faecal Pro (Qiagen), use of the Illumina platform, targeting of the V3/V4 regions, using the QIIME2 pipeline (or at least error modelling) in combination with the SILVA database. However, the research question, as well as reproducibility and consistency across studies should also be considered. To conclude, until standardisation of microbiome research occurs, significant consideration needs to be given to ensure correct interpretation of the literature and robust study design.

## Data Availability

The datasets used and/or analysed during the current study are available from the corresponding author on reasonable request.

## Abbreviations

16S-TRFLP: 16S terminal restriction fragment polymorphism
ASV: Amplicon sequence variant
DGGE: Denaturing gradient gel electrophoresis
dHPLC: Denaturing high-performance liquid chromatography
DNA: Deoxyribonucleic acid
dNTP: Deoxynucleoside triphosphate
emPCR: Emulsion polymerase chain reaction
FISH: Fluorescence in situ hybridization
InviMag Kit: InviMag Stool DNA Kit
MoBio Kit: MoBio Powersoil Bacterial DNA Kit
OTU: Operational taxonomic unit
PacBio SMRT: Pac Bio Single Molecule, Real-Time Sequencing
PCR: Polymerase chain reaction
PFGE: Pulse-field gel electrophoresis
PowerLyzer Kit: PowerLyzer PowerSoil Kit
QIAamp Kit: QIAamp DNA Stool Kit
QIIME: Quantitative Insights Into Microbial Ecology
qPCR: Quantitative polymerase chain reaction
RAPD: Random amplified polymorphic DNA
RDP: Ribosomal Database Project
RNA: Ribonucleic acid
Shotgun: Shotgun metagenomics
TGGE: Temperature gradient gel electrophoresis
TGS: Third generations sequencing
WGS: Whole genome sequencing
